# Chromatin balances cell redox and energy homeostasis

**DOI:** 10.1186/s13072-023-00520-8

**Published:** 2023-11-28

**Authors:** Tamaki Suganuma, Jerry L. Workman

**Affiliations:** Stowers Institute for Medical Research, 1000 E. 50th Street, Kansas City, MO 64110 USA

**Keywords:** Chromatin modification, Cellular homeostasis, Energy, Metabolism, Aging

## Abstract

Chromatin plays a central role in the conversion of energy in cells: alteration of chromatin structure to make DNA accessible consumes energy, and compaction of chromatin preserves energy. Alteration of chromatin structure uses energy sources derived from carbon metabolism such as ATP and acetyl-CoA; conversely, chromatin compaction and epigenetic modification feedback to metabolism and energy homeostasis by controlling gene expression and storing metabolites. Coordination of these dual chromatin events must be flexibly modulated in response to environmental changes such as during development and exposure to stress. Aging also alters chromatin structure and the coordination of metabolism, chromatin dynamics, and other cell processes. Noncoding RNAs and other RNA species that associate directly with chromatin or with chromatin modifiers contribute to spatiotemporal control of transcription and energy conversion. The time required for generating the large amounts of RNAs and chromatin modifiers observed in super-enhancers may be critical for regulation of transcription and may be impacted by aging. Here, taking into account these factors, we review alterations of chromatin that are fundamental to cell responses to metabolic changes due to stress and aging to maintain redox and energy homeostasis. We discuss the relationship between spatiotemporal control of energy and chromatin function, as this emerging concept must be considered to understand how cell homeostasis is maintained.

## Background

Maintaining cell homeostasis is fundamental to life. Metabolism controls the balance of acquisition and consumption of energy and is pivotal in maintaining cell homeostasis and cell responses to environmental changes during development, exposure to stress, and aging. Cell respiration and ATP synthesis are regulated by ATP utilization, and macromolecule biosynthesis is highly sensitive to the supply of energy [1]. Although metabolic pathways involve nutrient consumption and energy production, caloric restriction and reduction of basal metabolic rate can be beneficial for longevity [2, 3] due to reduced generation of reactive oxygen species (ROS) or decreased lipid oxidation [3–7]. (However, if an oxidative stress response is activated, production of ROS can also promote the expression of antioxidants [8]). Chromatin dynamics and modifications consume large amounts of metabolites [1, 3, 9–11] and rewire the cell’s metabolic status by controlling transcription [12–15]. Histones also store excess cell metabolites (e.g., acetylated histones store acetyl-CoA and methylated histones are methyl sinks) [16]. The catalytic activity of some chromatin modifiers is sensitive to the availability of oxygen in cells. During aging, chromatin regulation is impacted by changes in metabolism that increase the risk for cancer, diabetes, inflammation, and dementia [17]. Importantly, nucleosome density becomes low and nucleosome positioning becomes fuzzy in aged chromatin [3, 17]. Thus, aging and environmental changes that occur over a long period of time may impact chromatin function and gene expression.

In the study of chromatin’s role in cell homeostasis, a new concept has emerged from the observation that macromolecules including chromatin, RNA, and transcription activators are organized into membraneless subcellular compartments where they participate in biochemical reactions that are under spatiotemporal control [18, 19]. It has been suggested that this molecular assembly is driven by phase separation [20]. Phase separation is a physical process that creates distinct phases from a single mixture to minimize free energy [20–22], and enables specific components of the mixture to concentrate rapidly in one location [21]. Chromatin compaction/aggregation, which depends on NaCl and MgCl_2_ concentrations, is hypothesized to be a phase separation process [23, 24]. The generation and movement of macromolecules into subcellular compartments via phase separation may be essential to regulation of cell homeostasis by chromatin. Phase separation may be responsible for the robust increase in the concentration of RNA species observed in transcription regulation [18]. The yield of RNA species surrounding chromatin is now thought to be important in transcription activation. RNA biogenesis and processing sense changes in metabolism [25–27]. RNA species, particularly noncoding RNAs (ncRNAs), directly regulate transcription via different mechanisms [28]. However, how transcription regulation employs different types and quantities of RNAs is unclear. The amount of time required for generating the abundant RNAs that are rapidly recruited for transcription activation may be critical to the regulation of transcription and may be impacted by aging and other factors that affect cell metabolism. However, the roles of metabolism and nucleotide biogenesis in phase separation are unclear.

Here, we review the relationship between metabolism and chromatin dynamics while considering changes in metabolism and chromatin structure during aging. We also discuss emerging concepts regarding the regulation of chromatin modification and function by metabolic factors, noncoding RNAs, and phase separation.

## Main text

### Chromatin dynamics in aging

#### The expression levels of canonical histones and histone subtypes change during aging

**Fig. 1:**
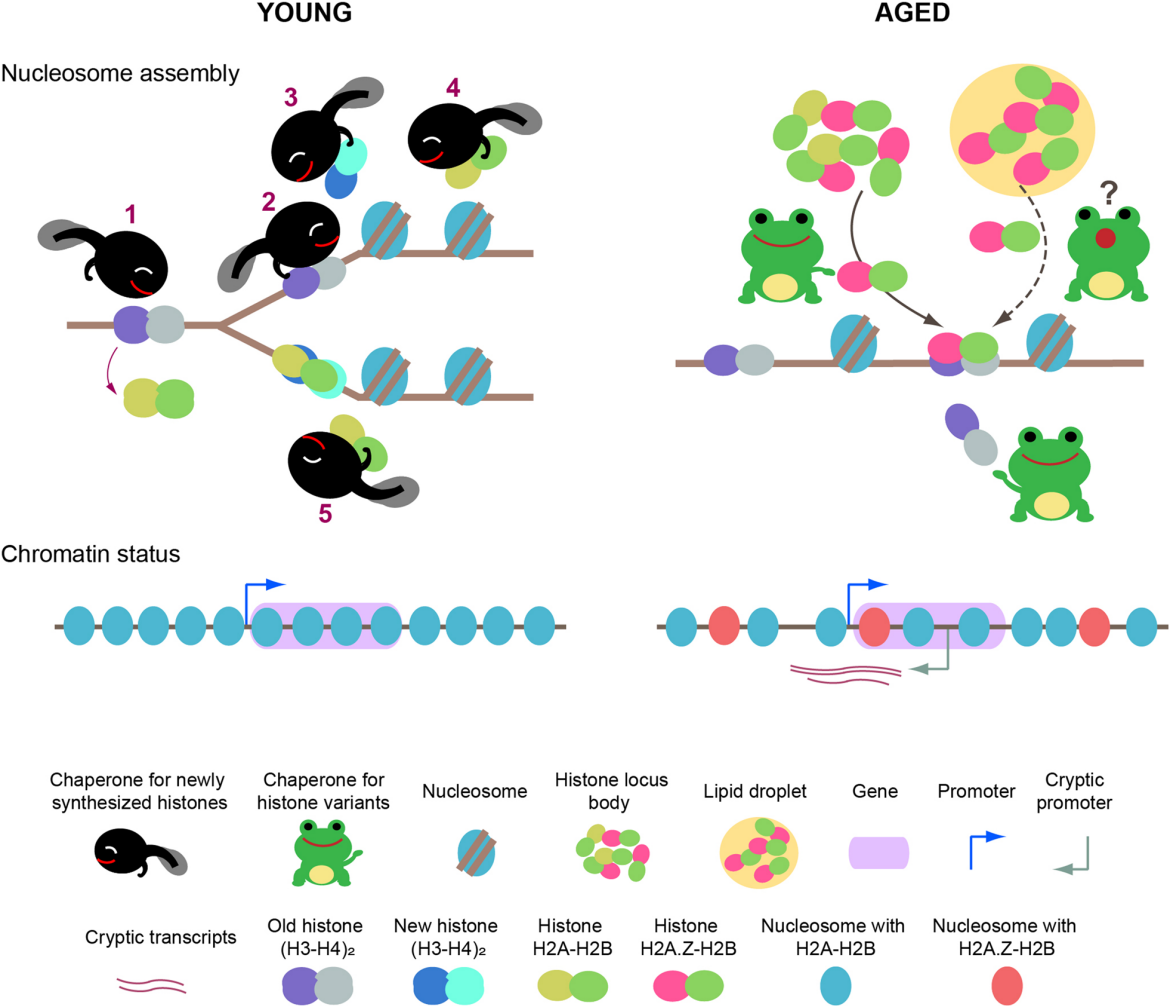
Relationship of replication-dependent and -independent nucleosome assembly to chromatin status during aging. In younger cells, histone chaperones deposit newly synthesized histones in replication-dependent nucleosome assembly and maintain chromatin with ordered nucleosome positioning throughout multiple cycles of transcription. Chaperones play roles in DNA packing at replication forks: 1‒disruption; 2‒transfer (recycling histones); 3‒transfer (newly synthesized histones); 4 and 5‒deposition/de novo assembly, are illustrated. However, in aged cells, the lengthened cell cycle reduces replication-dependent nucleosome assembly accompanied by incorporation of newly synthesized histones. Instead, other combinatory histone chaperones deposit histone variants through replication-independent nucleosome assembly, leading to ectopically and fuzzily positioned nucleosomes, causing cryptic transcription. Histones H2A, H2B, and H2A.Z are observed in histone locus bodies. H2A and H2AZ in lipid droplets may contribute to replication-independent nucleosome assembly

The basic subunit of chromatin is the nucleosome core particle. Nucleosomes contain a histone octamer wrapped with 147 base pairs (bp) of double-stranded DNA [9, 29]. The histone octamer consists of two central H3-H4 dimers flanked by two H2A-H2B dimers [9, 30]. Histone biosynthesis and nucleosome assembly can be replication-dependent or -independent [31]. Replication-dependent histones or “canonical histones”—H2A, H2B, H3, and H4—are encoded by genes that lack introns and contain a specific 40 bp sequence downstream of the stop codon that forms a consensus stem-loop structure at the 3’ end of the mRNA rather than a poly(A) tail [32, 33]. mRNA expression of these canonical histone genes, which are present in multiple copies in the genome, is replication dependent and high during S phase [32, 33] (Fig. 1). Core histone variants in humans include eight variants of H2A, six variants of H3, and two testis-specific variants of H2B [32]. Some histone variant genes contain introns. mRNAs of several histone variants (histone H3.3, H2A.Z (H2Av in *Drosophila melanogaster*), CENPA, macro-H2A, and H1.0) are polyadenylated and are replication independent (expressed throughout the cell cycle) [33]. An exception is H2A.X, which has mRNA that is polyadenylated in G0 and G1 but not in S phase [34].

The reversible nature of chromatin status enables epigenetic reprogramming, but chromatin structure is also modified by changes in metabolism during aging (Fig. 1). Micrococcal nuclease (MNase) sequencing revealed a significant reduction in nucleosome occupancy across the genome during replicative aging in budding yeast [35]. The lengthened cell cycle due to aging in yeast [36, 37] affects replication-dependent histone synthesis. The artificial overexpression of histones to compensate for reduced histone protein synthesis during replicative aging extends the replicative lifespan in yeast [38]. Ectopic histone H3 occupancy changes without significant changes in total H3 levels during aging in mice [39], although it is possible that this histone H3 includes both canonical and variant H3. Senescent cells synthesize less H3 and H4 than proliferating cells [40], but larger amounts of alternatively spliced histones [41]. These alternatively spliced (first coding exon to the second exon) histone H2A, H2B, and H4 subtypes may be counterparts of H3.3 [41]. The faster synthesis of replication-independent histones may be more suitable for stress responses than the slow synthesis of replication-dependent histones (Fig. 1). The alternative pool of histone subtypes may contribute to changes in chromatin structure during aging, as described below.

Histone storage, as opposed to suppressed synthesis, can regulate histone abundance. In *D. melanogaster*, clusters of histones H2A, H2B, and H2Av (the human H2A.Z homologue) have been found in histone locus bodies (HLBs) adjacent to Cajal bodies in nuclei [42]. Cajal bodies are membraneless spaces in nuclei formed due to liquid–liquid phase separation [42]. Cajal bodies play roles in RNA metabolism, formation of ribonucleoprotein particles, transcription, splicing, and telomere maintenance [43]. Excess histone H2A and H2Av synthesized during oogenesis are stored in lipid droplets in *D. melanogaster* [44] (Fig. 1). Disruption of the sequestration of Jabba, a protein that helps anchor histones to lipid droplets [45], increases the H2Av/H2A ratio and causes mitotic defects [44]. The nucleolus is another membraneless space for ribosomal RNA synthesis and ribosomal assembly [46]. Polyubiquitination of histone H2B at lysine 120 (H2BK120), which is degraded in the ubiquitination proteasome pathway, occurs in nucleoli [47]. Polyamines, which are positively charged and associate with anionic DNA and RNA at neutral pH [48], assist in nucleolar RNA production [49] and may contribute to nucleosome functions. Notably, S-adenosylmethionine (SAMe), an important methyl group donor for many metabolic reactions, is consumed in polyamine synthesis following decarboxylation to dcSAM [50]. Lipid stored in lipid droplets is utilized for energy production during starvation and for phospholipid synthesis to form membranes [51, 52]. Moreover, lipid droplets buffer excess lipids, prevent lipotoxicity and oxidative stress, and respond to DNA alkylation damage signaling [52, 53], and SAMe is highly consumed in phospholipid biosynthesis (as described below). Therefore, nucleoli and lipid droplets may regulate the expression of functional histones by sensing the abundance of methyl groups in cells.

#### Histone chaperones and remodeling complexes select histone subtype

Histone chaperones help select histones in the assembly or disassembly of nucleosomes, as each chaperone binds a different histone or combination of histones (Table 1). For example, Xenopus and human HIRA deposits histone H.3.3-H4 [54, 55], and yeast and human NAP1 deposits H2A-H2B and H2A.Z-H2B [56–58]. Human ASF1 transfers H3.1-H4 and H3.3-H4 dimers to CAF-1 and HIRA, respectively [59, 60]. Yeast Asf1 and Rtt109 H3K56 acetyltransferase rapidly replace histone H3 on the nucleosome in a replication-independent manner, although H3K56ac has a role as a mark of newly replicated chromatin [61, 62]. CAF-1 delivers newly synthesized H3-H4 dimers to the replication fork during S phase [63]. While DNA replication-dependent H3.1 is incorporated into chromatin in proliferating human cells [41], HIRA incorporates H3.3-H4 into the chromatin via its direct deposition or trafficking of non-chromatin-bound histones in senescent cells [41] (Fig. 1). Binding of HIRA with incorporation of newly synthesized H3.3 at the promoter of transcriptionally active genes enriched in H4K16ac is crucial for the steady-state abundance of H4K16ac in senescent cells [41]. The roles of chaperones in the packing of DNA at replication forks are illustrated in Fig. 1. The combinatory functions of chaperones and acetylated histones suggest that chaperones contribute to the response to metabolic status by providing different binding affinities for distinct histones.

**Table 1:** List of histone chaperones and corresponding bound histones during nucleosome assembly

Histone chaperone	Histone	Functions	Refernces
HIRA (Xenopus and humans)	H3.3-H4	Deposition of H3.3-H4	[50, 51]
NAP1 (yeast and Human)	H2A-H2B	H2A-H2B nuclear import and deposition	[52–54]
	H2A.Z-H2B,	Transfer of H2A.Z-H2B to SWR-C complex	
Nap1 (yeast)	H3-H4, H2A-H2B	Nucleosome assembly	[52, 53, 152]
CAF-1 (humans)	H3.1-H4	Deposition of H3.1-H4	[51]
FACT (humans)	H2A-H2B, H2A.X-H2B, H3-H4	Deposition of H2A-H2B, H2A.X-H2B, H3-H4	[153]
Chz1 (yeast)	H2A.Z-H2B	Incorporation of H2A.Z-H2B	[154]
ASF1 (yeast, Drosophila, and humans)	H3-H4	Transfer of H3-H4 and H3.1-H4 to RTT106 and CAF-1	[152, 153]
ASF1 (humans)	H3.1-H4	Transfer of H3.1-H4 to CAF-1	[56, 152, 153]
	H3.3-H4	Transfer of H3.3-H4 to HIRA	
ANP32E (humans)	H2A.Z-H2B	Removal of H2A.Z-H2B	[152, 156]
RTT106 (yeast)	H3-H4	Formation of (H3-H4)_2_	[157]

Examples of histone chaperones, each of which binds to a different histone or combination of histones. Xenopus and human HIRA (histone cell cycle regulation defective homolog A) deposits histone H.3.3-H4 [54, 55]; yeast and human NAP1 (nucleosome assembly protein 1) imports and deposits H2A-H2B or H2A.Z-H2B [56–58]; yeast Nap1 associates with H3-H4 and H2A-H2B [56, 57, 156]; human CAF-1 (chromatin assembly factor 1) deposits H3.1-H4 [55]; human FACT (facilitates chromatin transcription) deposits H2A-H2B, H2A.X-H2B, and H3-H4 [157]; yeast Chz1 incorporates H2A.Z-H2B [158]; yeast, *D. melanogaster*, and human ASF1 associates with H3-H4 [156, 159], H3.1-H4, and H3.3-H4 [60]; human ANP32E removes H2A.Z-H2B [156, 160]; and yeast Rtt106 forms (H3-H4)_2_ tetramer [161]

ATP-dependent chromatin remodelers catalyze histone deposition and removal. Histone H2A.Z is deposited into chromatin by the SWR-C/SWR1 chromatin remodeling complex (SWI2/SNF2-related 1, an INO80 type ATP-dependent remodeling complex), and is removed by the INO80 complexes in yeast [64–67]. Acetylated histones H4 and H2A catalyzed by the NuA4 histone acetyltransferase complex have been shown to be better substrates for SWR1-dependent exchange of H2A.Z-H4 dimers than their non-acetylated counterparts in experiments using purified proteins [62]. Interestingly, in *E. coli* the number of ATPases of RecQ helicase and the number of hydrolyzed ATP molecules used for the enzyme reaction are limited, as this ATPase loses its structure and function after hydrolyzing a certain number of ATP molecules [68]. This relationship suggests that the frequency of ATP hydrolysis in chromatin remodeling may also change during aging due to reduced cellular ATP levels. Loss of synchronization between histone synthesis, nucleosome assembly, and restructured chromatin by ATP-dependent chromatin remodeling [69] may increase the risk of DNA damage during aging. Deletion of *ISW2*, which encodes ISW2 ATPase in the ISW1 chromatin remodeling complex in yeast, derepresses genotoxic stress response genes upon calorie restriction and extends the replicative lifespan [70], suggesting that loss of *ISW2* increases stress resistance during aging.

Asf1 H3-H4 chaperone activity has been found to stimulate Set2 methyltransferase occupancy of the coding region of a highly transcribed gene in yeast [71]. Set2-catalyzed histone H3 lysine 36 mono/di/tri-methylation (H3K36me1/2/3) promotes the retention of existing histones and prevents histone exchange over the coding region [72]. Internal transcription from cryptic promoters is activated by loss of H3K36me [72]. In aged worms, cryptic transcription of a subset of genes is increased [73] (Fig. 1). Deletion of Set2 in yeast and of the Set2 homologue MET-1 in worm shortened replicative lifespan [73, 74]. Histone chaperones control nucleosome dynamics during elongation and contribute to histone exchange [75]. Increased histone exchange (i.e., in Set2 deficiency) and/or increasing the yield of cryptic transcripts that elevate the density of RNAs at promoters/enhancers which promote phase separation may reduce lifespan. (Phase separation is discussed in "Interactions between RNA and chromatin modulate energy homeostasis" section).

### Roles of chromatin modification in metabolism

#### Histone modifications in transcription and storage/recycling of metabolites in response to changes in metabolism

Glucose is the source of central precursor metabolites such as pyruvate, acetyl-Coenzyme A (acetyl-CoA), ATP, and 2-oxoglutarate in prokaryotes and eukaryotes [76]. These metabolites are generated via glycolysis, the tricarboxylic acid (TCA) cycle, the urea cycle, respiration, and oxidative phosphorylation [76, 77]. An emerging role of histones, in addition to their roles in the formation of nucleosomes and the regulation of transcription, is the storage of metabolites for recycling of histone modifications and for use in cellular processes and survival.

Acetyl-CoA is derived from catabolism of glucose, fatty acids, and amino acids. Acetyl-CoA is produced within the cytosol and mitochondria and pools in the cytosol and nucleus [78]. Cellular levels of acetyl-CoA influence global levels of histone acetylation [79]. In the presence of abundant carbon sources and oxygen, acetyl-CoA synthetase produces acetyl-CoA for histone acetylation in *S. cerevisiae*, which lacks ATP-citrate lyase (ACLY) [80]. In mammalian cells, ACLY predominantly catalyzes the conversion of citrate (from the TCA cycle) and coenzyme A to acetyl-CoA and oxaloacetate, which are used for synthesis of fatty acids and cholesterol in the cytoplasm, and also produces acetyl-CoA from citrate and CoA for nuclear acetylation [81, 82]. Under limited glucose and/or oxygen, nuclear acetyl-CoA is reduced due to requirement of acetyl-CoA for β-oxidation in mitochondria to synthesize ATP [78]. In these conditions, acyl-CoA synthetase short chain family member 2 (ACSS2) catalyzes acetate released from NAD^+^ -dependent histone deacetylation to synthesize acetyl-CoA for acetylation of histones H3K9, H3K27, and H3K56, which activates genes that synthesize lipids in human [83, 84]. In *S. cerevisiae*, the SAGA histone acetylase complex activates transcription of growth-promoting gene by H3K9 acetylation upon glucose repletion after starvation; however, upon glucose starvation the Rpd3 histone deacetylase complex deacetylates growth-promoting gene, leading to the release of acetate, which may participate to the SAGA-acetylation of genes that promote gluconeogenesis and lipid synthesis [85]. Thus, histones can store acetate for cell survival upon limited nutrition. Interestingly, fermentation of dietary fiber into butyrate by gut microbiota has been suggested to be a source of histone H4 acetylation across the genome except at promoter regions in mice epithelial cells [86]. It is unknown whether H4-specific histone acetyltransferases are abundant in the gut.

Short-chain acyl metabolic intermediates are substrates of histone lysine acylation including lysine propionylation, butyrylation, crotonylation, and β-hydroxybutyrylation [87]. These histone acylations have been found in highly transcribed genes [88, 89]. Acyl- and acetyl-lysines are recognized by the specific bromodomain of proteins with higher affinity [88, 90], suggesting that histone crosstalk involving recruitment of readers to lysine acylations or acetylations is also controlled by metabolic status [91].

Histones also function as a methyl sink. The methionine derivative SAMe is the major methyl donor for methylation in the cell. In *S. cerevisiae*, methylation of phosphatidylethanolamine (PE), a class of phospholipids present in membranes, is a major consumer of SAMe and promotes transsulfuration, which transfers sulfur from homocysteine to cysteine, leading to synthesis of cysteine, taurine, and glutathione (GSH) [16, 92]. GSH is a major antioxidant in yeast and mammals [16, 92]. When cho2 mutant cells, which abolish PE methylation, are cultured in synthetic media without amino acids and containing lactate, addition of methionine increases the methylation levels of histone H3 residues K36, K79, and K4 [16]. This histone methyl sink maintains transmethylation (conversion of SAMe to SAH) and transsulfuration, leading to prevention of oxidative stress [16]. Interestingly, SWI/SNF ATP-dependent chromatin remodeling complex transcriptionally regulates sulfur metabolism genes in yeast [15]. Loss of the Snf2 or Snf5 subunit results in impairment of cysteine biosynthesis during growth in rich media [15]. In mammals, cysteine is synthesized via the reverse transsulfuration pathway, which plays a central role in sulfur metabolism and redox homeostasis [92]. SAH levels in rat liver and cerebral cortex increase while SAMe levels in brain decrease with age [93]. Increased flux of the reverse transsulfuration pathway increases lifespan [94]. Misregulation of sulfur metabolism leads to increases in amyloid beta deposition that is a pathological feature of Alzheimer’s disease [92]. Methionine restriction extends lifespan in yeast and rodents [95–97]. Hence, the contributions of histones as methyl sinks and in transcriptional rewiring prevent sulfur toxicity and oxidative damage.

Histones can be phosphorylated at serine, threonine, or tyrosine residues. Histone phosphorylation plays important roles in DNA damage repair, transcription, and chromatin compaction [98]. Most histone phosphorylation is catalyzed by protein kinases that transfer the β- or γ-phosphate group from ATP to the hydroxyl group of these amino acids [99]. However, pyruvate kinase 2 (PKM2) in human (Pyk1 in yeast), which transfers a phosphate group from phosphoenolpyruvate (PEP) to ADP to generate pyruvate and ATP in the final step of glycolysis, also phosphorylates histone H3T11 [14, 100]. Pyk1 has been found in the serine-responsive SAM-containing metabolic enzyme (SESAME) complex, which contains serine metabolic enzymes (Ser33, Ser3, and Shm2), SAMe synthetases (Sam1 and Sam2), and an acetyl-CoA synthase (Acs2) [14]. SESAME delivers SAMe to the Set1 H3K4 methyltransferase complex. The interaction of SESAME with Set1 enables crosstalk between H3K4 methylation and H3T11 phosphorylation (H3pT11) by sensing glycolysis and glucose-derived serine metabolism [14]. In yeast, defects in H3pT11 modulate the nutritional stress response early in the chronological lifespan and result in longevity [101]. In humans, H3pT11 at promoter regions of *MYC* and *CCND1* by PKM2 is required for H3K9ac and subsequent expression of cyclin D1 and c-Myc [100]. Protein kinase C-related kinase 1 (PRK1) also phosphorylates H3T11 and activates androgen receptor-dependent transcription [102]. It will be interesting to see whether histones play a role in ATP or PEP storage in the regulation of metabolism.

#### Reduction and oxidation direct the activity of chromatin modifiers

90–95% of oxygen consumed by the body is utilized in mitochondria to generate ATP [103]. Oxygen availability influences metabolism which in turn affects post-translational protein modification. Furthermore, the availability of oxygen impacts histone methylation levels. JmjC histone lysine demethylases (KDMs) remove methyl groups from lysines within histone tails [104, 105]. The catalysts of JmjC are 2-oxoglutarate (2OG)-dependent dioxygenases (2-OGDs), which require molecular oxygen and iron (II) [104–106]. Oxygen sensing by 2-OGDs directly influences histone methylation status. The demethylase activity of KDM4E/JMJD2 on H3K9me3 was initially found to be dependent on oxygen availability in vitro [107]. It was subsequently found that demethylation of H3K9me2/3 and H3K36me2/3 by KDM4A/JMJD2 [108] is facilitated by high oxygen availability and is reduced upon hypoxia (1.0% O_2_) [104]. These roles were also observed for KDM5A/JARID1 toward H3K4me3/H3K36me3 and for KDM6A/UTX toward H3K27me3 [109, 110]. The EGLN1/PHD2, which catalyzes the post-translational formation of 4-hydroxyproline in hypoxia-inducible factor (HIF) alpha proteins, is also a 2-OGD [106]. Thus, the machinery of histone crosstalk [91] may be altered due to oxygen availability.

In humans, the sirtuin (SIRT) family consists of seven class III histone deacetylases (HDACs) [111]. SIRTs activate NAD^+^ in reactions with nucleophiles [112]. For example, in 19 reactions in pathways catabolizing glucose (glycolysis, conversion of pyruvate to acetyl-CoA, TCA cycle), NAD^+^ is involved in five reactions to capture a released electron and is further reduced to NADH [76, 113]. In yeast, Homologue of Sir Two (Hst1) suppresses genes involved in NAD^+^ biosynthesis [114, 115]. However, nicotinamide riboside, a NAD^+^ precursor that is converted to nicotinamide mononucleotide, elevates NAD^+^ levels and promotes Sir2-dependent repression of recombination and gene silencing, leading to extension of replicative lifespan regardless of calorie restriction in yeast [116]. Thus, NAD^+^ availability controls Sir2 activity, and Sir2 feeds back to NAD^+^ biogenesis via its gene regulation.

### Interactions between RNA and chromatin modulate energy homeostasis

#### Metabolism connects chromatin modifiers to mRNA translation

There are direct connections between chromatin modifying complexes and the mRNA translation machinery in response to metabolic changes. The double-stranded RNA-dependent protein kinase (PKR) was initially found to be activated by its binding to viral double-stranded RNA and regulates the innate immune response [117]. In mammalian cells, PKR activates c-Jun N-terminal kinase (JNK) and insulin receptor substrate 1 and inhibits translation initiation by phosphorylation of translation initiation factor eIF2α in response to nutrient excess, resulting in inhibition of insulin signaling [118]. PKR inhibits translation of the iron-responsive mRNA through eIF2α phosphorylation [119]. In mammals and *Drosophila*, the Ada Two-A-containing (ATAC) acetyltransferase complex activates JNK signaling and transcription of JNK target genes upon osmotic stress [120]. However, innate PKR activity is suppressed by the association of the ATAC and molybdopterin (MPT) synthase, leading to transcription suppression of JNK target genes and promotion of translation [120, 121]. Thus, association of ATAC with MPT synthase switches the activities of transcription to translation in response to metabolic signals. MPT synthase regulates sulfur amino acid metabolism [121], which is essential for mRNA processing [25, 122]. tRNA thiolation is required for efficient translation of genes, expression of which is important for translation and cell growth under limited nutrients in yeast [27]. Downregulation of tRNA thiolation during sulfur starvation promotes compensatory expression of enzymes involved in methionine, cysteine, and lysine biosynthesis [27]. Dysregulation of sulfur amino acid metabolism and transsulfuration creates sulfur toxicity and ROS, triggering neurological dysfunctions including Alzheimer’s disease [92, 123, 124]. Thus, transcriptional regulation and modification of tRNA may be coordinated during nutritional stress.

#### Direct or indirect association of chromatin modifiers with ncRNAs in transcription

Noncoding RNAs function as transcriptional regulators. Long noncoding RNAs (lncRNAs) transcribed from a minor promoter upstream of human *dihydrofolate reductase* (*DHFH*) suppress transcription from the downstream major *DHFR* promoter [125]. The suggested mechanism is triplex formation between single-stranded RNA and double-stranded DNA at the promoter. DNA:DNA:RNA triplex formation was also found at the *SPHK1* promotor, by probing with an in vitro triplex capture assay and EMSA [126]. The lncRNA *KHPS1* is transcribed antisense to *SPHK1* and binds the *SPHK1* promoter. This triplex further recruits p300/CBP, leading to increases in H3K27ac and H3K9ac and in the accessibility of the transcription factor E2F1, resulting in activation of *SPHK1* transcription [126]. RNA:DNA duplexes are also found in epigenetic silencing machinery. The *fragile X mental retardation 1* (*FMR1*) gene is silenced by hybridization of a transcribed CGG-repeat tract of the 5’ UTR *FMR1* mRNA to the complementary CGG-repeat of the *FMR1* gene [127]. H3K9me2 at *FMR1* promoter is observed; however, it is unknown whether *FMR1* mRNA recruits chromatin modifiers that directly change H3K9me2 levels, or whether this is a consequence of gene silencing by RNA:DNA duplex formation alone. Additional studies are needed to elucidate whether ncRNA alone regulates transcription or whether the association of ncRNAs with chromatin modifiers underlies the transcription regulation (discussed below).

The association of silencing factors and lncRNA—such as the association of polycomb-repressive complex 1 (PRC1) and PRC2 with lncRNA *Xist* and *HOTAIR*—provides examples of gene silencing by lncRNA. Histone H2AK119 mono ubiquitination (H2AK119ub1) catalyzed by PRC1 initiates polycomb-mediated transcriptional repression and stabilizes H3K27me3 deposition by PRC2 in embryonic stem cells (ESCs) [128, 129]. hnRNPK was found to bind Xist RNA polycomb interaction domain (XR-PID) of *Xist* RNA, and non-canonical PRC1 (vPRC1), PCGF3/5/RING1 complex, leading to *Xist*-dependent H2AK119ub1 and *Xist*-mediated gene silencing [130]. Synthetically tethering hnRNPK to XistΔXR-PID is sufficient for H2AK119ub1 deposition, and tethering hnRNPK mutant that loses the ability to bind RING1 (H2AK119ub1 catalyst) eliminates H2AK119ub1 deposition [130]. Therefore, binding of hnRNPK to both *Xist* and PCGF3/5/RING1 is required for *Xist*-mediated gene silencing [130]. It has been proposed that RNP granules are formed by liquid–liquid phase separation [131] (described in the next section). Thus, an interesting question is whether *Xist* RNA mediates polycomb interactions with super-enhancers in these structures. The lncRNA *HOTAIR* is transcribed from the *HOXC* locus and suppresses *HOXD* by deposition of PRC2-mediated H3K27me3. A 5’ domain of *HOTAIR* RNA binds to PRC2 whereas a 3’ domain of *HOTAIR* binds the LSD1/CoREST/REST complex [132]. *HOTAIR* prevents gluteal adipocyte development by the interaction of *HOTAIR* RNA with PRC2 which silences genes that are involved in adipocyte lineage in iliofemoral adipose tissue [133]. The gluteal adipose tissue is one of major factors determining waist-to-hip ratio associated with obesity-related metabolic disorders [134]. In this regulation, PRC2 is not required for *HOTAIR* to bind chromatin [135]. *HOTAIR*-mediated transcriptional repression in PRC2-depleted breast cancer cells was also observed [136]. Thus, *HOTAIR* may play a role in the restriction of the PRC2-dependent H3K27me3 loci.

Genome sequencing and RNA-sequencing in mouse cortical neurons showed that RNA polymerase II (Pol II) at H3K4me1-marked enhancers bi-directionally transcribes enhancer RNAs (eRNAs) which are synthesized at adjacent genes and positively correlate with the mRNA levels of these genes upon activation of calcium-dependent signaling [137]. This observation suggests that eRNA and Pol II at enhancers activate transcription. Most of these eRNAs are not polyadenylated and are likely to mediate interactions between the enhancer and a promoter adjacent to the gene [137]. Estrogen receptor transcription factor rapidly and robustly binds the promoter of its target genes upon 17β-estradiol (E2) ligand treatment and activates transcription of not only protein-coding genes but also noncoding RNAs (ncRNAs) by redistribution of active forms of all three RNA polymerases, including intergenic region as observed using global run-on and sequencing (GRO-seq) in human breast cancer cell lines [138]. The clusters of enhancers, which include large numbers of ncRNAs and other eRNAs are called “super-enhancers” (SEs), and have been linked to a phase separation model of transcription regulation [18] (described in the next section). It will be important to understand whether metabolism modulates the functions of ncRNAs by altering their quality or quantity.

#### Role of phase separation in transcription regulation

**Fig. 2:**
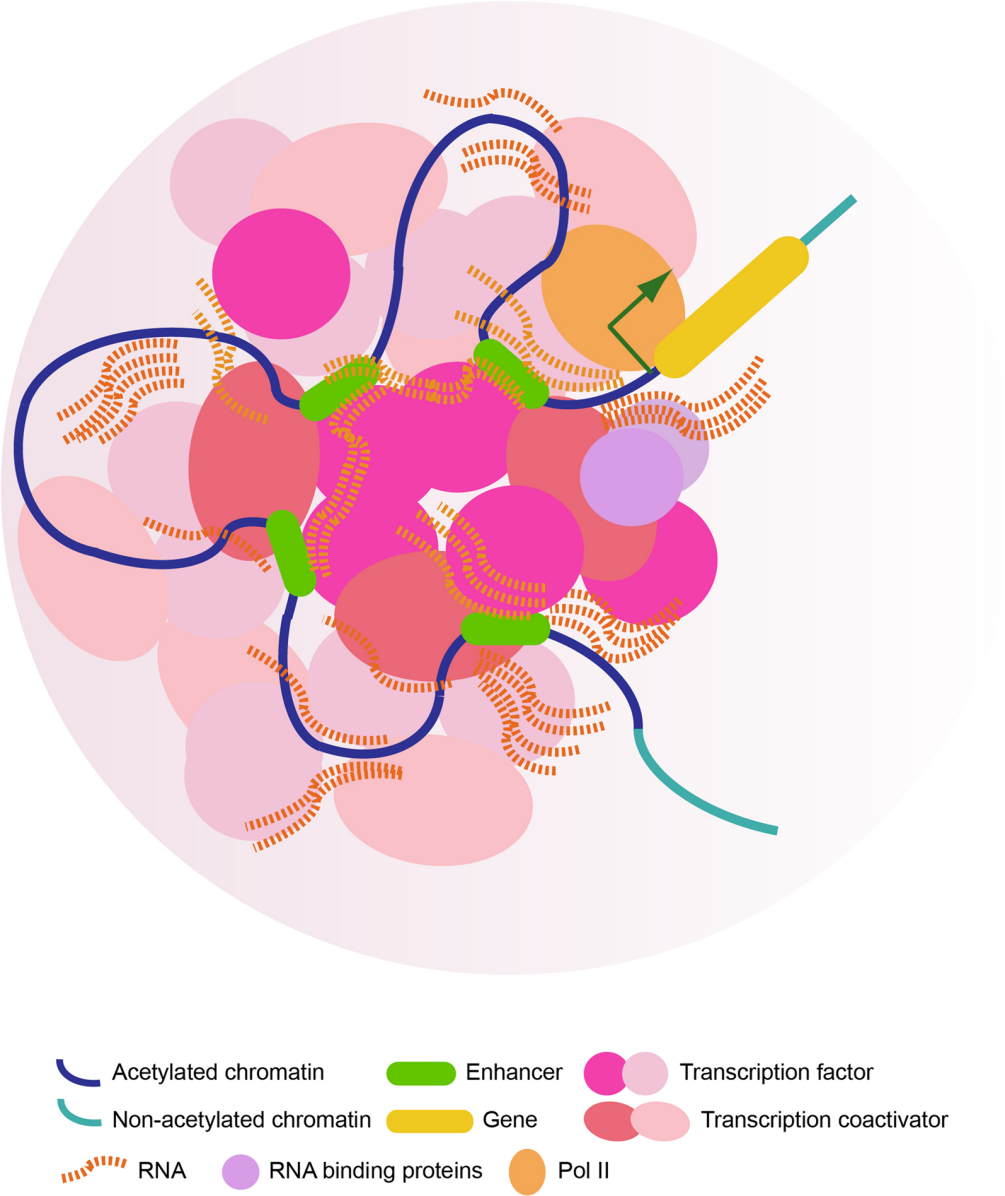
The high density of macromolecules at super-enhancers causes phase separation. Phase separation is proposed as the mechanism by which transcription factors, co-activators, and enhancer RNAs are condensed in clustered enhancers (super-enhancers, SEs) with highly acetylated nucleosomes in transcription activation. The figure depicts phase separation of phosphorylated RNA-polymerase II condensates mediated by promoter-associated RNAs and their binding proteins in transcription activation

An emerging concept is that liquid–liquid phase separation (LLPS) participates in genome organization and remodeling [23, 137, 138]. Phase separation is a process that creates distinct dense and diluted phases from a single mixture [20–22], and allows components to become rapidly concentrated in one place [21]. Non-membrane-bound compartments in cells such as nucleoli, Cajal bodies, paraspeckles, and lipid droplets are compartments formed via phase separation [139, 140]. Phase separation has been proposed to explain the observation of clusters, of both transcription activators and hundreds or thousands of RNAs including bi-directional eRNA into super-enhancers [18, 137, 138, 141, 142] (Fig. 2). The increase in eRNA transcripts also contributes to transcription activation. Super-enhancer-associated eRNAs induced by activation of Toll-like receptor 4 signaling in macrophages activate genes that drive innate immunity, indicating that eRNA activates transcription of signaling-specific genes [142]. Biochemical analysis of mESCs revealed that RNA-binding proteins such as paraspeckle component 1 guide Pol II to the formation of transcription condensations and lead to phosphorylation and release of Pol II for transcription activation through their binding to multivalent RNA molecules [143] (Fig. 2). Stimulation of tumor necrosis factor alpha (TNFα) rapidly redistributes transcription activators, including NF-κB and p300, to the enhancers [144]. Acetylation of the p65 subunit of NF-κB at Lys310 by p300 upon TNFα treatment [144, 145] is directly recognized by bromodomains of bromodomain containing 4 (BRD4), and this interaction is required for NF-κB transactivation [146]. BRD4 but not H3K27ac is essential for creation of TNFα-induced super-enhancers, which are enriched in BRD4, p65, and H3K27ac, to rapidly promote transcription of inflammatory responsive genes [144]. Super-enhancer formation was proposed to result from phase separation in a model of transcription control [18].

Acetylation on lysines has been thought to neutralize their positive charge and reduces interaction of histones with negatively- charged DNA [9, 87, 147, 148], providing transcription factors access to their binding sites [9]. This observation raises the question of whether increased binding of bromodomain-containing transcription factors preserves acetylated histones at nucleosomes of super-enhancers from further binding of bromodomain-containing chromatin remodelers such as SWI/SNF [9], to avoid remodeling chromatin during rapid stress. Moreover, it is unclear whether the biosynthesis of nucleotides [149] which drive increases in enhancer RNA at super-enhancers is specifically promoted by inflammatory signaling. Phase separation may contribute to chromatin reorganization at super-enhancers as chromatin aggregation may be relevant to liquid–solid phase separation in cell nuclei [23].

Highly positively charged LCDR/IDR (low complexity or intrinsically disordered region) of the chromobox 2 (CBX2/M33) subunit of PRC1 causes chromatin compaction of PRC1 in *Drosophila* and mice [150]. Induction of higher expression levels of CBX223KRA mutant, which carries at 23 lysine and arginine residues mutated to alanine in its compaction region, showed less repressive transcription activity than CBX WT in mESC [151]. H2AK119ub levels, which are catalyzed by PRC1, in CBX2^23KRA^ was not shown. Importantly, *Cbx2*^*23KRA/23KRA*^ mice as well as *Cbx2*^*−/−*^ mice display defects in patterning of the body axis [151]. Multivalency caused by IDR of proteins is a principle of LLPS [152]. For example, the C-terminal prion-like domain of TAR DNA binding protein (TARDBP/TDP-43), which is a typical IDR, is essential for TDP-43 phase transitions [152]. N-terminal tail domains of histones are disordered [23]. Further studies of the effects of intrinsically disordered histones and chromatin associations of proteins on phase separation, and of the effect of fuzzy nucleosomes during aging on phase separation are needed.

## Concluding remarks and future perspectives

Depending on the requirements of cell signaling and metabolism, chromatin utilizes canonical histones or different histone subtypes which are synthesized at different periods. Histone chaperones select canonical histones and/or histone subtypes in response to various stimuli or for different biological processes. However, how histone chaperones sense metabolic status is still unclear. The dual functions of histones for metabolite storage and in chromatin functions make histones and chromatin themselves look like a metabolic pathway. There are still questions about the fate of metabolite-storage in histones and these metabolites. If intergenic regions of histone H4 acetylation function as acetate storage, it must be re-considered whether modifications of nucleosomes are direct regulators of nucleosome functions including transcription. Importantly, the interplay between chromatin modifiers and metabolism increases with the availability of oxygen, which provides cell energy via oxidation, and the goal of this interplay is prevention of the generation of excess reactive oxygen species.

RNA molecules directly participate in chromatin functions and transcription through their association with chromatin or chromatin modifiers. The effects of metabolism and aging on the quality of those RNA species (e.g., intact RNAs, ncRNAs, snRNAs, and so on) must be elucidated. Accumulated observations of high yields of enhancer RNAs at super-enhancer regions raise questions about how nucleotides are produced for transcripts and enhancer RNAs upon a rapid stimulus. More studies of the mechanisms connecting transcription and translation are needed.

Recent concepts of phase separation in the regulation of super-enhancers recalls the activation of stress-activated kinases (SAPKs), such as the activation of MAP kinase (MAPK) and JNK pathways [153, 154]. Ste20-type kinases that activate MAPK/JNK signaling sense K^+^, Na^+^, and Cl^−^, which in turn control cell volume [155]. Ste20-kinases are conserved from prokaryote to eukaryote [155]. Consequently, phosphorylation/activation of JNK upon osmotic stress activates JUN transcription factor, activating transcription of JNK target genes [120]. Changing osmolality or K-Cl and Na–K-2Cl cotransporters and the duration of (rapid) stimuli that are responded to by SAPKs may contribute to phase separation and may be a part of mechanism of super-enhancer creation. It will be interesting to investigate whether signaling pathways that create super-enhancers link to K-Cl and Na–K-2Cl transporters. The flow rate of molecules into highly dense compartments might be a key element in the creation of super-enhancers. In SAPK pathways, phosphorylation and dephosphorylation regulate transcriptional activation of target genes. It is yet to be elucidated whether post-translational modifications (PTMs) of macromolecules in super-enhancers are synchronized and whether those PTMs enable macromolecules to deposit with high flux and play a role in the activation and termination of super-enhancer-dependent transcription. The involvement of PTMs could suggest a role for aging in the regulation of super-enhancers.

## Data Availability

The datasets in the current study are available from the corresponding authors on reasonable request.
